# A Rice B-Box Protein, OsBBX14, Finely Regulates Anthocyanin Biosynthesis in Rice

**DOI:** 10.3390/ijms19082190

**Published:** 2018-07-27

**Authors:** Da-Hye Kim, Sangkyu Park, Jong-Yeol Lee, Sun-Hwa Ha, Jun-Gu Lee, Sun-Hyung Lim

**Affiliations:** 1 National Institute of Agricultural Science, Rural Development Administration, Jeonju 54874, Korea; kimdh143@jbnu.ac.kr (D.-H.K.); psk2779@korea.kr (S.P.); jy0820@korea.kr (J.-Y.L.); 2 Department of Genetic Engineering and Graduate School of Biotechnology, Kyung Hee University, Yongin 17104, Korea; sunhwa@khu.ac.kr; 3 Department of Horticulture, College of Agriculture & Life Sciences, Chonbuk National University, Jeonju 54896, Korea; jungu@jbnu.ac.kr

**Keywords:** anthocyanin, B-box protein, *Oryza sativa*, transcription factor

## Abstract

Anthocyanins are responsible pigments for giving attractive colors of plant organs and nutraceutical benefits of grains. Anthocyanin biosynthesis is known to be regulated by transcription factors and other regulatory proteins. In rice (*Oryza sativa*), the R2R3 MYB transcription factor (TF) OsC1 and a bHLH TF, OsB2, were previously reported to control anthocyanin biosynthesis in vegetative tissues and seeds, respectively; however, the regulatory mechanisms of the anthocyanin biosynthesis by TFs remain largely unknown. In this study, we identified OsBBX14, a homolog of *Arabidopsis thaliana* B-box domain protein 22 (AtBBX22), and investigated its function. The transcript level of *OsBBX14* was high in pigmented rice seeds and gradually increased as the seeds matured. The ectopic expression of *OsBBX14* in *Arabidopsis* resulted in a dramatic increase in anthocyanin accumulation in its seedlings. Using a steroid receptor-based inducible activation system, OsBBX14 and OsHY5 were found to directly activate *OsC1* or *OsB2* in an independent or collaborative manner. Yeast two hybrid revealed that the second B-box domain of OsBBX14 physically interacts with the bZIP domain of OsHY5. These results suggest that the anthocyanin biosynthesis in rice is induced and finely tuned by OsBBX14 in collaboration with OsHY5.

## 1. Introduction

Rice (*Oryza sativa* L.), together with maize (*Zea mays*) and wheat (*Triticum aestivum*), is an important staple for a large part of the world’s population. Most rice varieties have white (or beige)-colored grains, although some varieties have brown, red, or black seeds. The red or black color of the rice grain is determined by the accumulation of proanthocyanidins (PAs) or anthocyanins, respectively [1,2]. Several studies have reported that pigmented rices contain elevated level of various phytochemicals that is beneficial to human health [3,4], which is of significant interest to both rice breeders and consumers.

Anthocyanin biosynthesis is catalyzed by multiple enzymes in the flavonoid biosynthetic pathway, which has been extensively characterized in a variety of plant species [5,6,7]. The structural genes encoding enzymes in the anthocyanin biosynthetic pathway are regulated by a ternary transcriptional regulatory complex, MBW, composed of MYB, basic helix-loop-helix (bHLH), and a WD40 repeat (WDR) [8,9]. In rice, the R2R3-MYB transcription factor (TF), OsC1, activates anthocyanin biosynthesis in most organs except the pericarp [10]. OsB2 and Rc are bHLH TFs involved in anthocyanin and proanthocyanidin (PA) biosynthesis, respectively, in a tissue-specific manner [1,2]. Several studies show that WDR proteins interact with different R2R3-MYBs and bHLHs to form various MBW complexes, which play roles in anthocyanin and PA accumulation in vegetative tissues and developing seeds [5,11]; however, little is known about the WDR genes involved in anthocyanin and PA biosynthesis in rice.

Anthocyanin biosynthesis is affected by a variety of environmental factors, including light, temperature, and nutrient (N/C) balance [12,13,14]. Studies in *Arabidopsis thaliana* and other plant species have revealed the stimulatory effect of light on anthocyanin production via the increased expression of the R2R3-MYB and structural genes [15,16]. In *Arabidopsis*, ELONGATED HYPOCOTYL 5 (HY5) is considered as a key signaling player in photomorphogenesis, and directly activates the expression of the R2R3-MYB TF, production of anthocyanin pigment 1 (*PAP1*) and anthocyanin biosynthesis genes, chalcone synthase (*CHS*), and chalcone isomerase (*CHI*) by binding to their promoters [14,17]. Several studies have therefore shown that genes controlling HY5 can positively and negatively regulate anthocyanin biosynthesis [18,19].

B-box proteins (BBX), which contain one or two B-box domains at their N-terminal region, function as TFs and have roles in plant growth and development [18]. The BBX proteins are classified into five subfamilies based on their phylogeny and domain compositions in *Arabidopsis* and rice [20,21]. In *Arabidopsis*, BBX proteins in the subfamily IV group contain two tandem repeat B-box domains in their N-terminal region, and function as negative or positive regulators of the light-stimulated anthocyanin biosynthetic pathway [19]. Some of these group IV proteins, including AtBBX21, AtBBX22, AtBBX24, and AtBBX25, have been reported to physically interact with AtHY5 [17,22,23,24,25]; AtBBX21 and AtBBX22 act as coactivators of AtHY5, whereas AtBBX24 and AtBBX25 repress its TF activity. The *bbx21* and *bbx22* mutant seedlings accumulate less anthocyanin than wild type (WT) seedlings, while the *bbx24* and *bbx25* mutants contain more anthocyanin than the controls [22,23].

In this study, we identified a novel transcriptional regulator of the anthocyanin biosynthetic pathway in rice, OsBBX14. Our results indicate that OsBBX14 regulates the accumulation of both anthocyanin and chlorophyll during photomorphogenesis in transgenic *Arabidopsis* plants. We also demonstrate that OsBBX14 can induce *OsC1* expression by coregulating and physically interacting with OsHY5 in rice. Taken together, our findings suggest that OsBBX14 acts as a fine regulator of anthocyanin biosynthesis in rice.

## 2. Results

### 2.1. Identification of B-Box Genes from White and Black Rice Seeds

To identify novel regulators of anthocyanin biosynthesis in rice seeds, we performed an RNA-seq analysis of Ilmi (IM), Heugnam (HN), and Heugjinju (HJJ) rice harvested at 15 days after pollination (DAP). This analysis led to the identification of a zinc finger gene that was highly expressed in the black rice seeds (HN and HJJ), which was designated as *OsBBX14* according to Huang’s nomenclature [20]. OsBBX14 belongs to subfamily IV of the BBX proteins as it contains two B-box domains but no CCT domain. Sequence alignments showed that OsBBX14 has the conserved zinc finger motifs of B-box I (CX_2_CX_8_CX_2_DXAXLCX_2_CDX_3_H) and B-box II (CX_2_CX_8_CX_2_DX_3_LCX_2_CDX_3_H) at its N-terminal region, as well as small conserved motifs, motif 6 (M6), motif 7 (M7), and the VP pair, in the central region, and a nuclear localization signal (NLS) at its C-terminal end (Figure S1). The two conserved B-box domains, small conserved motifs, and NLS are found to be not only in the subfamily IV BBX proteins in dicot plants such as *Arabidopsis*, apple (*Malus × domestica*), tomato (*Solanum lycopersicum*), and soybean (*Glycine max*), but also in monocot species such as barley (*Hordeum vulgare*) and maize (Figure S1), suggesting that these proteins are highly conserved among the angiosperms.

**Figure 1:**
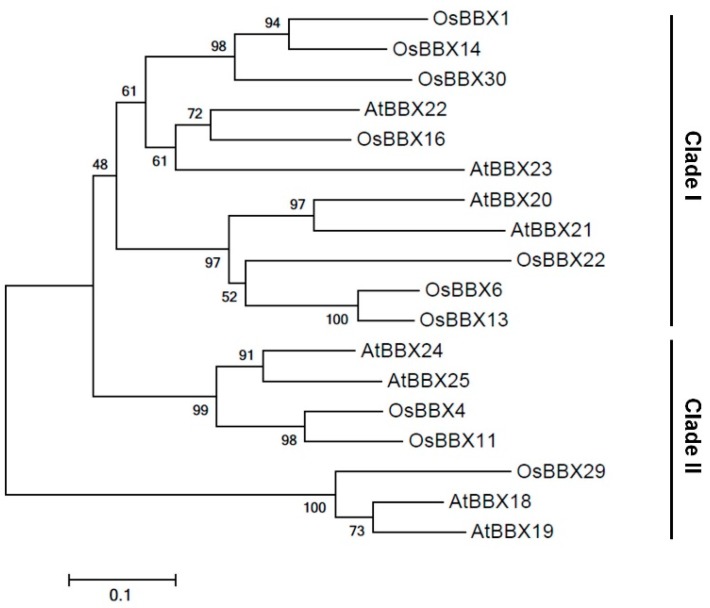
Phylogenetic tree of the BBX proteins of subfamily IV in *Arabidopsis* and rice. The numbers next to the nodes are bootstrap values from 1000 replications. The tree is drawn to scale, with branch lengths in the same units as those of the evolutionary distances that were used to infer the phylogenetic tree (scale bar, 0.1 amino acid substitutions per site). The deduced amino acid sequences were retrieved from the DDBJ/EMBL/GenBank databases. AtBBX18 (AT2G21320), AtBBX19 (AT4G38960), AtBBX20 (AT4G39070), AtBBX21 (AT1G75540), AtBBX22 (AT1G78600), AtBBX23 (AT4G10240), AtBBX24 (AT1G06040), and AtBBX25 (AT2G31380) are *Arabidopsis thaliana* proteins; OsBBX1 (Os01g0202500), OsBBX4 (Os02g0606200), OsBBX6 (Os02g0646200), OsBBX11 (Os04g0493000), OsBBX13 (Os04g0540200), OsBBX14 (Os05g0204600), OsBBX16 (Os06g0152200), OsBBX22 (Os06g0713000), OsBBX29 (Os09g0527900), and OsBBX30 (Os12g0209200) are *Oryza sativa* proteins.

A phylogenetic analysis of the subfamily IV BBX proteins in *Arabidopsis* and rice grouped these proteins into two clades, suggesting that it is most likely due to changes in the C-terminal regions [26]. OsBBX14 was clustered within the same clade as AtBBX22 (AT1G78600) (Figure 1). Subfamily IV BBX proteins that regulate anthocyanin biosynthesis and photomorphogenesis are divided into two different clades according to their sequence homology; one clade includes AtBBX22 (hereinafter referred to as clade I), and the other includes AtBBX24 (hereinafter referred to as clade II). The BBX proteins of clade I are involved in the control of de-etiolation and hypocotyl growth, and positively regulate anthocyanin production, whereas the BBX proteins of clade II are involved in antagonistic interactions that negatively regulate seedling photomorphogenesis and anthocyanin biosynthesis [19,22,23,24,25]. The highly conserved nature of the BBX domains, especially within the subfamily IV group, suggests that their functional diversity is most likely due to the changes in the C-terminal region [26]. As a member of clade I, OsBBX14 might function as a positive regulator of seedling photomorphogenesis and anthocyanin biosynthesis in rice.

### 2.2. Temporal Expression of OsBBX14 and Other Regulatory Genes in Developing Rice Seeds

**Figure 2:**
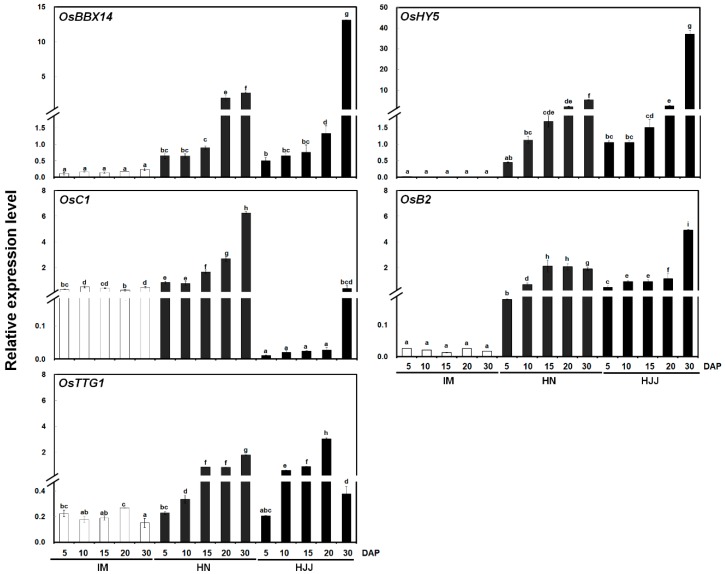
Expression of *OsBBX14* and putative anthocyanin regulatory genes in nonpigmented (IM) and black (HN and HJJ) rice varieties during seed maturation. The numbers along the *x*-axis reflect the days after pollination (DAP). Results represent mean values ± SD from three biological replicates. Different letters above the bars indicate significantly different values (*p* < 0.05) calculated using two-way ANOVA followed by a Duncan’s multiple range tests.

The temporal expression patterns of *OsBBX14* during rice seed development were analyzed using a quantitative real-time polymerase chain reaction (qPCR) (Figure 2). The transcript levels of *OsBBX14* were very low in the nonpigmented IM rice seeds, but were high in both HN and HJJ rice seeds. During seed maturation, the transcript levels of *OsBBX14* gradually increased in both nonpigmented and black rice seeds. In the black rice, the transcript level of *OsBBX14* dramatically increased at 15 DAP, which coincided with the initiation of seed pigmentation, and gradually increased until 30 DAP (Figure 2).

In addition, we performed a qPCR analysis to investigate the transcript levels of anthocyanin biosynthesis regulators in rice plants, including genes encoding basic leucine zipper (bZIP) TF (*OsHY5*), R2R3-MYB TF (*OsC1*), bHLH TF (*OsB2*), and WDR TF (*OsTTG1*). The expression of *OsHY5* and *OsB2* was significantly upregulated in HN and HJJ rice seeds and gradually increased during seed maturation, compared to nonpigmented IM rice seed. Interestingly, the *OsC1* expression levels were the highest in HN, but the lowest in HJJ rice seeds. The transcript levels of *OsTTG1* in nonpigmented IM rice seed were a similar regardless seed maturation stages, but those in HN and HJJ rice seeds were upregulated during seed maturation. The expression pattern of *OsHY5* and *OsB2* genes at seed maturation stages was similar to that of *OsBBX14*, in both of the HN and HJJ. Taken together, these results suggest that high expression levels of *OsBBX14* are associated with high levels of *OsHY5* and *OsB2* transcript and pigmentation of black rice seeds during their maturation.

### 2.3. Subcellular Localization and Transcriptional Activation Activity of OsBBX14

**Figure 3:**
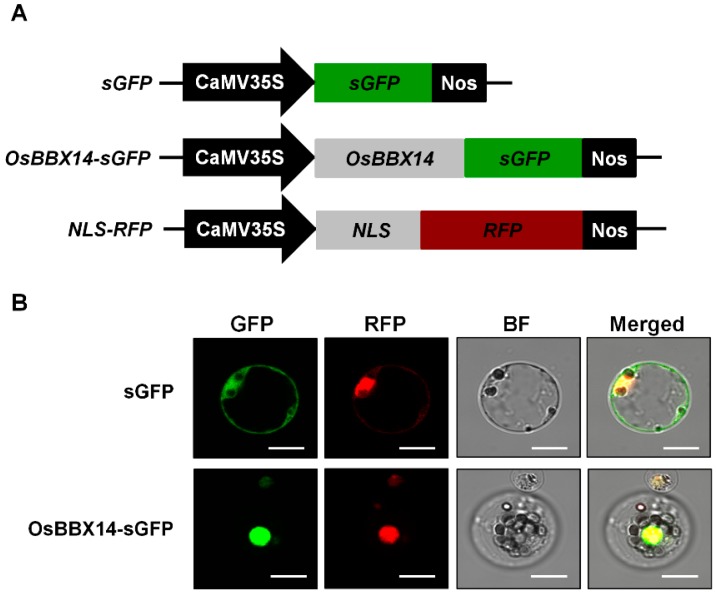
Subcellular localization of OsBBX14 in rice leaf protoplasts. (**A**) The three constructs used in this experiment are shown schematically. OsBBX14-sGFP, OsBBX14 fused to sGFP; NLS-RFP, nuclear localization signal fused with RFP; (**B**) in vivo targeting of OsBBX14 in rice protoplasts. The fluorescence distribution of the control sGFP and the OsBBX14-sGFP fusion protein are shown under GFP fluorescence (green), the nuclear localization signal is represented by the RFP fluorescence (red). Images are representative of the protoplasts expressing fusion proteins at 16 h after transformation. Scale bars, 10 µm.

To provide further evidence for the potential role of OsBBX14 in transcriptional regulation, an OsBBX14-sGFP fusion protein was expressed in rice leaf protoplasts under the control of the cauliflower mosaic virus (CaMV) 35S (CaMV35S) promoter. As shown in Figure 3, the fluorescence of the control-sGFP protein was distributed throughout the cell, whereas the OsBBX14-sGFP fusion protein was exclusively located in the nucleus, indicating that OsBBX14 exhibits nuclear localization.

**Figure 4:**
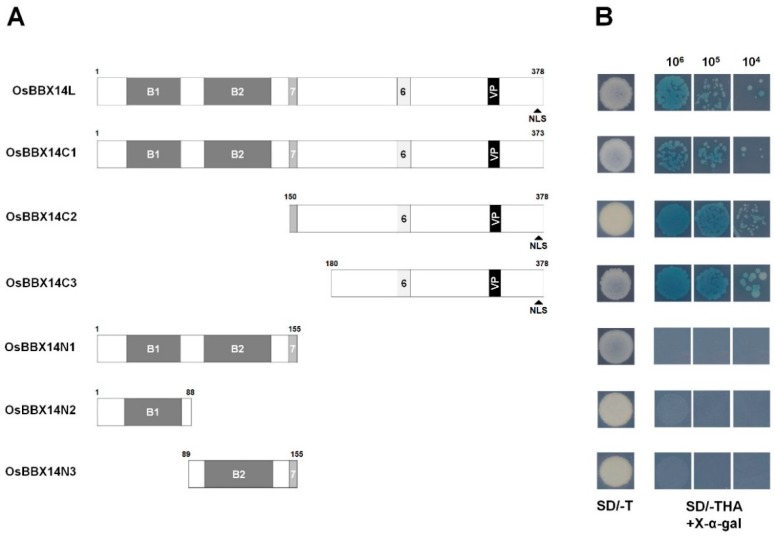
Transactivation assay of OsBBX14. (**A**) Schematic diagrams of various constructs used in the transcriptional activity assay. (**B**) Transactivation analysis in yeast. Amino acid positions are labeled in the diagrams. B1, first B-box; B2, second B-box; 7, Motif 7; 6, Motif 6; NLS, nuclear localization signal; SD/−T, minimal medium lacking Trp; SD/−THA+X-α-Gal, minimal medium lacking Trp, His, and Ade but containing 20 mg/mL X-α-Gal; VP, VP domain; 10^6^, 10^5^, and 10^4^ indicate the number of cells dotted on each plate of media.

To analyze whether OsBBX14 has transcriptional activation activity, seven constructs containing full length (OsBBX14L) and partially truncated OsBBX14 proteins (OsBBX14C1, OsBBX14C2, OsBBX14C3, OsBBX14N1, OsBBX14N2, and OsBBX14N3) were fused in-frame to the GAL4 DNA-binding domain (GAL4-BD) of the pGBKT7 vector, after which their transcriptional activation activity was assayed in yeast (Figure 4). These constructs were transformed into the yeast strain AH109, then screened on both synthetic dropout (SD) media lacking tryptophan (SD/−T) and a triple dropout medium, which is SD media lacking tryptophan, histidine, and adenine (SD/−THA), supplemented with X-α-Gal. The transformants containing OsBBX14L grew well on the selection medium SD/−THA and turned blue in the presence of the chromogenic substrate X-α-Gal, indicating that OsBBX14 had strong activation activity. Among the partial clones of OsBBX14, the yeast transformants containing OsBBX14N1, OsBBX14N2, and OsBBX14N3, respectively, which lacked the C-terminal and central regions of the protein, did not grow on the selective medium and did not turn blue under the same conditions. In contrast, the partial clones, OsBBX14C1, OsBBX14C2, and OsBBX14C3, which lacked the N-terminal region of the protein, were able to grow on the selection medium. This suggests that the C-terminal and central region of OsBBX14 is required for its transcriptional activation activity. Taken together, these results indicate that OsBBX14 is a nucleus-localized transcription factor with a transcriptional activation activity, and its C-terminal is a functional region for the transcriptional activation.

### 2.4. Overexpression of OsBBX14 in Arabidopsis Caused an Anthocyanin Accumulation and Short Hypocotyl Length

**Figure 5:**
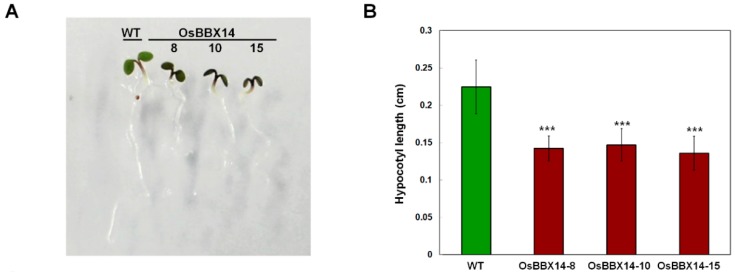

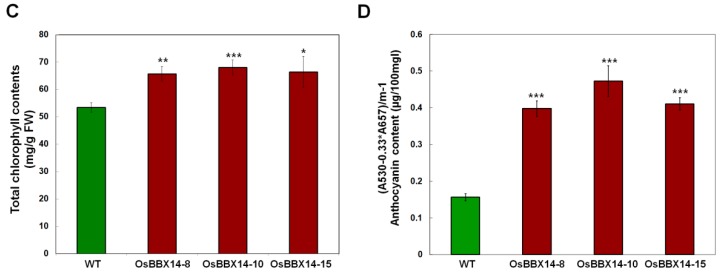
Ectopic expression of *OsBBX14* affects photomorphogenesis in *Arabidopsis*. (**A**) Representative seedlings of wild type (WT) *Arabidopsis* plants and three independent *OsBBX14*-expressing transgenic *Arabidopsis* plants grown under fluorescent light for seven days. (**B**) Hypocotyl length. (**C**) Chlorophyll contents. (**D**) Anthocyanin contents. Three biological replicates were averaged and statistically analyzed using Student’s *t*-tests (* *p* < 0.05, ** *p* < 0.01, *** *p* < 0.001). Bars indicate the standard deviation (SD) of the mean.

To investigate the function of OsBBX14, we generated transgenic *Arabidopsis* lines expressing *OsBBX14* under the control of the CaMV35S promoter. We selected three of the 15 independent *OsBBX14* transgenic plants obtained by floral dipping for further analysis. As shown in Figure 5, *OsBBX14* transgenic *Arabidopsis* plants had shorter hypocotyl lengths, darker green leaves, and accumulated more anthocyanin than the WT plants. Under normal fluorescent light (FL) conditions, the hypocotyls of seven-day-old seedlings were 34–40% shorter in the *OsBBX14* transgenic lines than the WT plants, while the chlorophyll content of the *OsBBX14* transgenic *Arabidopsis* plants was 23–27% higher than the WT plants. In addition, the *OsBBX14* transgenic lines showed a 2.5- to 3-fold increase in anthocyanin content when compared with the WT plants. These results indicated that OsBBX14 plays an important role in photomorphogenesis, regulating the accumulation of anthocyanin and chlorophyll as well as the length of the hypocotyl.

### 2.5. Overexpression of OsBBX14 Induced the Expression of Anthocyanin Biosynthesis Regulatory Genes in Arabidopsis

**Figure 6:**
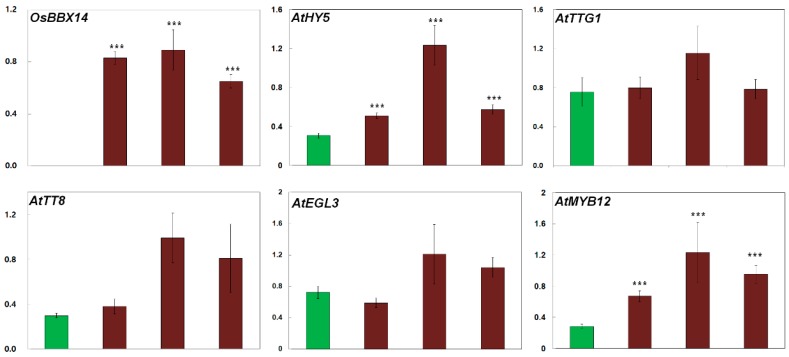

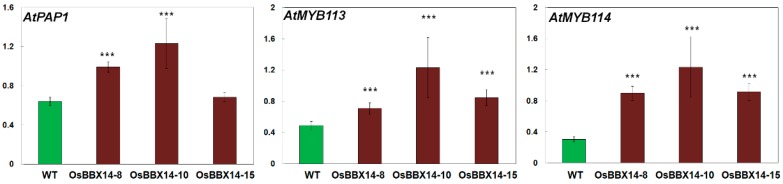
Expression analysis of anthocyanin biosynthesis regulatory genes in transgenic *Arabidopsis* plants expressing *OsBBX14*. All results represent the mean value ± SD from three biological replicates. *** indicate values that significantly differ from the expression level in WT at *p* < 0.001 according to Student’s *t*-tests.

Anthocyanin biosynthesis is known to be controlled by several TFs mediating the activation of the promoters of anthocyanin biosynthesis genes [8,9]. We performed a qPCR analysis to examine the expression levels of endogenous regulatory genes in the leaves of the transgenic *Arabidopsis* plants, including genes encoding basic leucine zipper (bZIP) TF (*AtHY5*), WDR TF (*AtTTG1*), bHLH TFs (*AtTT8* and *AtEGL3*), and R2R3-MYB TFs (*AtMYB12*, *AtPAP1*, *AtMYB113*, and *AtMYB114*). The transgenic expression of *OsBBX14* caused a significant upregulation of *AtHY5*, *AtMYB12*, *AtPAP1*, *AtMYB113*, *AtMYB114*, and *AtTT8*, but had no effect on the expression of *AtTTG1* and *AtEGL3* (Figure 6). These results suggest that OsBBX14 regulates the expression of *AtHY5* and the R2R3-MYB-type TF genes in *Arabidopsis* seedlings.

### 2.6. OsBBX14 Directly Activates the Expression of the Anthocyanin Regulatory Genes

To further explore the role of OsBBX14 as a regulator of anthocyanin biosynthesis in rice, a steroid receptor-based inducible activation system was developed as described previously [27]. In this system, a TF fused to the hormone-binding domain of the glucocorticoid receptor (GR) is sequestered in a complex of chaperone proteins in the cytoplasm, which maintains its inactive state. Upon treatment with a synthetic steroid hormone, dexamethasone (DEX), the transcription factor is released from the complex and enters the nucleus to regulate the expression of its downstream target genes. Coupled with a protein synthesis inhibitor, cycloheximide (CHX), this steroid-mediated activation system enables the identification of the direct targets of transcription factors in plants [27].

**Figure 7:**
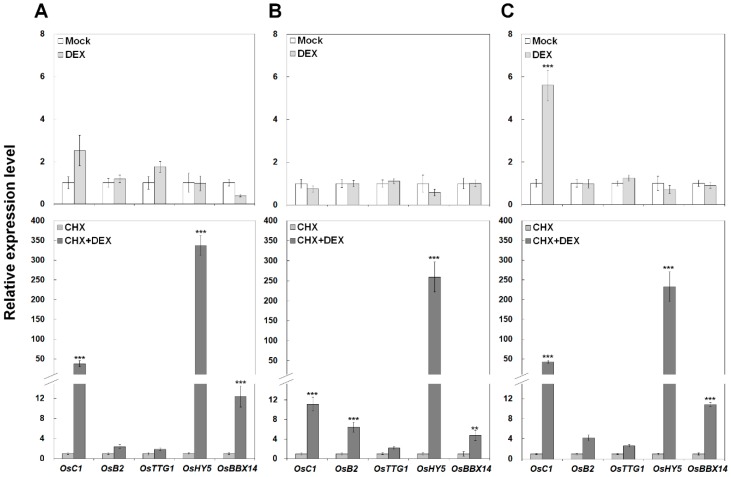
qPCR analysis of the regulation of anthocyanin regulators by OsBBX14 and OsHY5 in rice protoplasts. (**A**) Transcript levels in rice protoplasts transformed with *OsBBX14-GR*. (**B**) Transcript levels in rice protoplasts transformed with *OsHY5-GR*. (**C**) Transcript levels in rice protoplasts cotransformed with *OsBBX14-GR* and *OsHY5-GR*. Protoplasts were treated with DEX (a synthetic hormone used to activate the GR-bound transcription factor; upper row) alone or in combination with CHX (a protein synthesis inhibitor; lower row). The data represent the mean of three biological replicates (with three technical replicates for each biological sample). Asterisks denote significance according to Student’s *t*-tests (** *p* < 0.01, *** *p* < 0.001), where DEX-treated samples are compared with the mock, and DEX+CHX-treated samples are compared with CHX-treated samples.

OsBBX14 was fused to the hormone-binding domain of the GR and constitutively expressed under the control of the CaMV35S promoter in rice leaf protoplasts (Figure 7A). After CHX and/or DEX treatment, the induced expression of the anthocyanin regulatory genes was examined by qPCR. Particularly, primer sets for *OsBBX14* and *OsHY5* were designed at 3′-UTR region of their mRNA sequences to discriminate between the expression of endogenous and exogenous genes. Upon DEX treatment, the OsBBX14-GR protein slightly induced the expression of *OsC1*; however it did not activate the expression of other genes. While, after treatment with CHX and DEX simultaneously, OsBBX14-GR dramatically upregulated the expression of *OsC1*, *OsHY5*, and *OsBBX14*, resulting in approximately 50-, 350-, and 12-fold increment, respectively. In several studies, HY5 has been reported to play a role in anthocyanin biosynthesis [14]. Additionally, our study showed that *AtHY5* was activated by the ectopic expression of *OsBBX14* in *Arabidopsis* (Figure 6). Thus, to clarify the regulatory role of OsHY5 in anthocyanin biosynthesis, rice protoplasts were transfected with *OsHY5-GR*. As shown in Figure 7B, OsHY5-GR did not directly activate any genes under DEX treatment; however, after a simultaneous treatment with CHX and DEX, the expression of *OsC1*, *OsB2*, *OsHY5*, and *OsBBX14* was increased approximately 11-, 6-, 260-, and 4-fold, respectively. These results show that individual OsBBX14-GR and OsHY5-GR could directly activate endogenous *OsC1*, *OsHY5*, *OsBBX14*, and/or *OsB2*. To confirm the synergic regulation of OsBBX14 and OsHY5, rice protoplasts were cotransfected with *OsBBX14-GR* and *OsHY5-GR* (Figure 7C). Under the DEX treatment, the transcription level of *OsC1* was noticeably upregulated approximately 5.5-fold; however, under the combined DEX and CHX treatment, the transcription of *OsC1*, *OsHY5*, and *OsBBX14* was dramatically increased approximately 50-, 230-, and 11-fold, respectively, by the cotransfection of *OsBBX14-GR* and *OsHY5-GR*. Overall, the regulatory genes were significantly induced under the simultaneous treatment with CHX and DEX, which implies the unknown repressor related in anthocyanin biosynthesis might be suppressed by CHX treatment. The induced expression of the endogenous *OsBBX14* and *OsHY5* by the transfection with *OsBBX14-GR* and *OsHY5-GR*, respectively, suggests that these two genes could be subject to autoregulation. Collectively, these results indicate that OsBBX14 and OsHY5 could directly activate the regulatory genes of anthocyanin biosynthesis such as *OsC1* and *OsB2* in an independent or collaborative manner.

### 2.7. Protein–Protein Interactions between OsBBX14 and OsHY5

**Figure 8:**
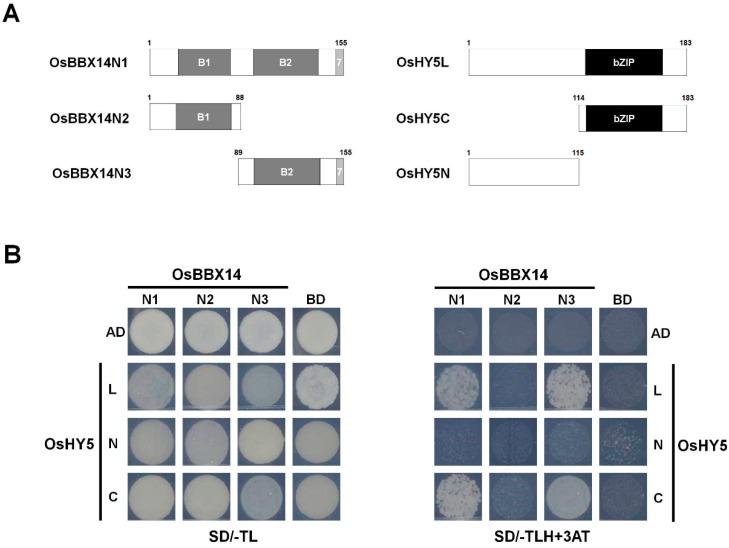
Physical interaction between OsBBX14 and OsHY5. (**A**) Schematic diagram of the constructs used in the Y2H experiment. The amino acid positions of these fragments are numbered. B1, first B-box; B2, second B-box; bZIP, bZIP domain. (**B**) Protein-protein interactions between OsBBX14 and OsHY5 revealed using a Y2H analysis. SD/−TL, minimal medium lacking Trp and Leu; SD/−TLH+3AT, minimal medium lacking Trp, Leu, and His, but containing 10 mM 3-amino-1,2,4-triazol (AT). N1, N2, and N3 indicate OsBBX14 N-terminal fragments, OsBBX14N1, OsBBX14N2, and OsBBX14N3, respectively. L, N, and C indicate OsHY5 whole protein (OsHY5L), its N-terminal (OsHY5N), and its C-terminal regions (OsHY5C), respectively. AD and BD indicate activation domain and binding domain, respectively.

To investigate the interaction between OsBBX14 and OsHY5 at the molecular level, we performed a yeast two-hybrid (Y2H) assay. When OsBBX14 was fused with GAL4-BD, its transcriptional activation was detected; however, it disappeared when the C-terminal 223 amino acids of OsBBX14 were deleted (Figure 4). To identify the domains responsible for the OsBBX14-OsHY5 interaction, Y2H assays were performed using partially truncated OsBBX14 proteins (OsBBX14N1, OsBBX14N2, and OsBBX14N3) and either the whole OsHY5 protein (OsHY5L) or its N-terminal or C-terminal regions (OsHY5N and OsHY5C, respectively). As shown in Figure 8, OsBBX14N1, containing the B-box 1 and B-box 2 domains, strongly interacted with OsHY5C, which harbored the bZIP domain. OsBBX14N2, containing only the B-box 1 domain, was unable to interact with OsHY5. Notably, OsBBX14N3, containing only the B-box 2 domain, was able to interact with OsHY5C. These data indicate that B-box 2 of OsBBX14 and the bZIP domain of HY5 are required for the interaction of these two proteins.

## 3. Discussion

Anthocyanin biosynthesis is regulated by MBW complexes in numerous plant species, including *Arabidopsis*, apple, petunia, and maize [8,9]. MBW complex activity is controlled by various environmental conditions, including light, temperature, and the nutrient balance [12]. Novel modulators have been reported to regulate the transcription of MBW complex genes to affect the plant development [28]. In rice, several studies revealed that OsC1 and OsB2 affect anthocyanin accumulation in the vegetative tissues and the pericarp, respectively; however, no novel modulators of anthocyanin biosynthesis have been reported in rice to date.

### 3.1. OsBBX14 Is a B-Box Protein with Transcriptional Activity

Through the RNA-seq analysis of the rice seeds, we identified that the level of *OsBBX14* transcripts was higher in pigmented rice seeds than in nonpigmented rice seeds. The deduced amino acid sequence of OsBBX14 contained well conserved domains and motifs of the subfamily IV BBX proteins (Figure S1). The subfamily IV BBX proteins are divided into two clades containing positive and negative regulators of photomorphogenesis. Generally, the N-terminal regions of the proteins in the two clades are well conserved, while their C-terminal regions are diverse, suggesting that their functional diversity is most likely due to changes in the C-terminal region [26]. There is some evidence that the substitution of the conserved Asp residues in the two BBX domains into an Ala residue results in a dysfunctional protein, which disrupts its ability to regulate and interact with HY5 [22,29]. In the C-terminal region, the VP pair is critical for the interaction of the BBX proteins with COP1, which regulates the dark-specific degradation of proteins [30]. Recent studies confirmed the role of the C-terminal region of BBX proteins in photomorphogenesis by swapping between the domains of AtBBX21 and AtBBX24, positive and negative regulators of this process, respectively [31]. Transgenic *Arabidopsis* plants producing AtBBX21 with the C-terminal region of AtBBX24 had reduced anthocyanin contents and longer hypocotyls at high intensities of light, while transgenic plants producing AtBBX24 with the C-terminal of AtBBX21 showed an enhanced accumulation of anthocyanin and shorter hypocotyls, resembling the phenotype of plants ectopically expressing *AtBBX21* in *Arabidopsis*. In addition, transgenic *Arabidopsis* plants producing AtBBX24 containing the C-terminal region of AtBBX21, which includes the M6 region, showed strong hypersensitivity to light similar to transgenic *Arabidopsis* plants with AtBBX21, while transgenic plants producing AtBBX24 containing the C-terminal of AtBBX21, which lacks M6, showed no hypersensitivity to light. Thus, the C-terminal region containing the M6 region of AtBBX21 is a positive regulator of photomorphogenesis, and the M6 region determines the function of the C-terminal region in light signaling.

We found that OsBBX14 has transcriptional activation activity in yeast cells (Figure 4). The OsBBX14 fragment lacking the M7 and NLS domains of the C-terminal region could still activate gene transcription in yeast cells. The C-terminal region of OsBBX14 contains acidic amino acid residues and proline-rich domains, which are known to be important for activating transcription [32]. Taken together, these results suggested that the C-terminal region of OsBBX14 plays an important role in photomorphogenesis and the regulation of transcription.

### 3.2. OsBBX14 Modulates the Expression of Anthocyanin Biosynthesis Regulators by Interacting with OsHY5

Several studies revealed that the clade I and clade II of subfamily IV BBX proteins in *Arabidopsis* and rice play positive and negative roles in mediating the photomorphogenesis response, respectively [19]. AtBBX21, AtBBX22, and AtBBX23 are clustered in the clade I of subfamily IV and are involved in the accumulation of anthocyanin and the positive regulation of photomorphogenesis, whereas AtBBX24 and AtBBX25 are clade II proteins involved in the negative regulation of de-etiolation and the hypocotyl shade avoidance response. In *Arabidopsis*, AtBBX21 and AtBBX23 physically interact with AtHY5 and enhance its transcriptional regulation of downstream gene expression, whereas AtBBX24 and AtBBX25 physically interact with AtHY5 and thereby interfere with its binding to the promoters of its target genes [22,31,33]. The ectopic expression of *OsBBX14* in *Arabidopsis* and the transfection experiments in rice protoplasts revealed that, like other clade I subfamily IV BBX proteins, OsBBX14 promotes the expression of *AtHY5* in *Arabidopsis* and *OsHY5* in rice protoplasts, respectively (Figure 6 and Figure 7). Furthermore, the qPCR analysis of the regulatory genes in the rice seeds (Figure 2) demonstrated that the expression patterns of *OsHY5* during seed maturation coincided with that of *OsBBX14* in the nonpigmented and pigmented rice seeds. Taken together these results, it confirmed that OsBBX14 promotes the expression of *OsHY5* in pigmented rice seeds, which is consistent with the results of AtBBX21 and AtBBX23 from *Arabidopsis* [31,33]. A high similarity in the sequence and functionality of the proteins belonging to the clade I of subfamily IV BBX indicates that these proteins are well conserved in dicotyledonous and monocotyledonous plants. The ectopic expression of *OsBBX14* in *Arabidopsis* resulted in an enhanced anthocyanin and chlorophyll content compared with the WT plants (Figure 4). Previous studies have reported that AtBBX23 regulates the transcription of *AtHY5* and promotes the expression of light-induced genes, including *CHS* and *EARLY LIGHT-INDUCIBLE PROTEIN 2* [33]. The positive regulators AtBBX21 and AtBBX23 modulate downstream gene expression by coordinating with AtHY5 [31,33]. Recent studies have shown that the second B-box domain of AtBBX21 directly binds to the T/G-box of the *AtHY5* promoter to activate its expression, which in turn serves to increase the level of AtHY5 protein in the cell and modulate its transcriptional activity to promote photomorphogenesis [34]. It has been known that HY5 directly binds to either G-box or ACE box of the promoters of R2R3 MYB TFs or induces the expression of structural genes related to anthocyanin biosynthesis in combination with other MYB TFs [14,34]. In addition, HY5 promotes several genes involved in chlorophyll biosynthesis and chloroplast biogenesis through direct bindings to the ACE box in their promoters [35,36]. Similar to the results of previous studies, the higher levels of *OsHY5* and *OsB2* expression were observed in the pigmented rice seeds in which *OsBBX14* was highly activated (Figure 2), suggesting that *OsHY5* is positively regulated by OsBBX14, and which is likely to further activate *OsC1*, *OsB2*, and *OsTTG1* for anthocyanin accumulation in the seeds of pigmented rice during maturation. Unlike *OsB2* and *OsTTG1*, *OsC1* was promoted only in the seeds of pigmented rice cultivar HN during maturation, implying that there would be additional factors involved in this regulation with cultivar-specific manner. The gene expression analysis using the DEX-inducible activation system in protoplast showed that OsBBX14-GR independently induced the expression of *OsC1* and *OsHY5*, however *OsB2* and *OsTTG1* expression was not induced (Figure 7), which suggests that *OsC1* and *OsHY5* could be direct targets of individual OsBBX14 protein. Interestingly, individual OsHY5-GR protein was also predominantly activated by endogenous *OsHY5*. This allows speculation that *OsHY5* could be subject to autoregulation, which can be supported by several previous reports demonstrating that AtHY5 is autoregulated by direct binding to its own promoter [37,38]. The independent *OsBBX14-GR* transfection and *OsBBX14-GR* and *OsHY5-GR* cotransfection showed similar patterns and levels of the regulatory genes expression, and *OsB2* expression was not induced in both cases. These phenomena imply that only the two proteins may not be sufficient to properly regulate the pathway. Therefore further study is needed to identify additional components involved in this regulation.

The yeast two hybrid analysis revealed that the second B-box domain of OsBBX14 physically interacts with the bZIP domain in the C-terminal region of OsHY5 (Figure 8), which supports our suggestion that anthocyanin production in the pigmented rice seeds can be induced and finely tuned by the regulatory function of OsBBX14 in collaboration with OsHY5. The C-terminal regions of BBX proteins have recently been reported to play important roles in photomorphogenesis [31]. Future research on the roles of the C-terminal region of the rice BBX proteins may provide a more clear insight into the regulatory mechanism of anthocyanin accumulation in rice seeds.

## 4. Materials and Methods

### 4.1. Plants Materials

Rice seeds were obtained from the Agricultural Genetic Resources Center at the National Institute of Agricultural Science (Jeonju, Korea). The following three rice cultivars were used, categorized according to their pericarp color; white: IM; black: HJJ and HN. These rice cultivars were grown in the field at the rice experimental station of National Institute of Agricultural Science. The seeds of *Arabidopsis thaliana* Columbia-0 (Col-0) were subjected to cold treatment at 4 °C for three days and then grown in soil under long-day conditions (LD; 16-h 100 µmol m^−2^·s^−1^ light/8-h dark) at 22 °C before transformation.

### 4.2. Transcriptome Profiling

Total RNAs were isolated from seeds of three rice cultivars at 15 DAP using the Illumina TruSeq RNA Sample Prep Kit (Illumina, San Diego, CA, USA). Six libraries, comprising two biological replicates each of IM, HJJ, and HN, were sequenced using the Illumina HiSeq 2000 system. Clean reads were obtained by removing the adapter sequences and any low-quality reads, which were identified using the SolexaQA package (v2.5, Illumina, San Diego, CA, USA). Clean reads were mapped using the RNA-seq alignment algorithm implemented in Bowtie2 software [39] to the transcripts of *Oryza sativa* MSU release 7 from Phytozome V.9 [40] allowing all aligning with a maximum of two mismatches. The number of mapped clean reads for each gene was counted and then normalized with DESeq package in *R* to avoid bias due to different of sequencing amount.

### 4.3. RNA Isolation and qPCR

The qPCR was conducted using RNA from rice seeds at various developmental stages, including 5, 10, 15, 20, and 30 DAP. Total RNA was extracted from the rice seeds using the Fruit-mate for RNA Purification solution (Takara, Otsu, Japan) and the Plant RNA Purification Reagent (Invitrogen, Carlsbad, CA, USA) as described previously [41], then purified using the FavorPrep™ Plant Total RNA Mini Kit (Favorgen, Changzhi, Taiwan). Total RNA was prepared from rice protoplasts and *Arabidopsis* seedlings using the TRIzol reagent (Invitrogen) and purified using the FavorPrep™ Plant Total RNA Mini Kit (Favorgen), according to the manufacturer’s instructions. The cDNAs were synthesized from 2 μg of total RNA using an amfiRivert cDNA Synthesis Platinum Master Mix (GenDEPOT, Barker, TX, USA).

The qPCRs were performed using AccuPower 2x Greenstar qPCR Master Mix (Bioneer, Daejun, Korea) and the BioRad CFX96 Detection System (Bio-Rad Laboratories, Hercules, CA, USA), according to the manufacturer’s instructions. The expression levels of the target genes were normalized using the ubiquitin (*UBI*) gene for rice and the elongation factor 1α (*EF1α*) gene for *Arabidopsis* as internal references. The gene-specific primers used for the qPCR analysis are listed in Table S1. Three biological replicates and three technical replicates were performed for each sample.

### 4.4. Bioinformatics Analysis

The nucleotide sequence, deduced amino acid sequence, and open reading frame (ORF) of *OsBBX14* were subjected to a BLAST analysis using the National Center for Biotechnology Information (NCBI) website (http://www.ncbi.nlm.nih.gov). A structural analysis of the deduced protein was conducted using the ExPASy Molecular Biology Server (http://cn.expasy.org/tools/). Multiple sequence alignments were performed using ClustalW [42]. A phylogenetic tree was constructed using the neighbor-joining method [43] in MEGA version 6 software [44].

### 4.5. Subcellular Localization Analysis and Plant Transformation

The ORF of *OsBBX14* was amplified using an *OsBBX14*-specific primer set (OsBBX14-F: 5′-CACCATGTCGCCTCCTCCTCCACCATAT-3′; OsBBX14-R: 5′-TTATTGCCTCCGGCGTTTGGAGGTGGTGGC-3′). The obtained PCR fragments were then cloned into the pENTR/D-TOPO vector (Invitrogen) and sequenced in triplicate to validate the DNA sequences.

For the subcellular localization analysis, the ORF of *OsBBX14* was amplified using the gene-specific primer sets (p326-OsBBX14-F/R), which were introduced into an *Xba*I-digested p326-sGFP plasmid using the In-Fusion HD Cloning Kit (Takara). The resultant p326-OsBBX14-sGFP plasmid was sequenced to confirm the absence of errors during PCR amplification. The plasmids were introduced into rice protoplasts prepared from rice leaf tissues using a polyethylene glycol-mediated transformation, as described by Kim et al. [45]. The expression of the p326-OsBBX14-sGFP construct was determined 16–20 h after transformation, and images were captured using fluorescence confocal microscopy (Leica TCS SP8; Leica Microsystems, Wetzlar, Germany).

For the transformation of *Arabidopsis*, the ORF of *OsBBX14* in the pENTR/D-TOPO vector (Invitrogen) was incorporated into the Gateway destination vector pB7WG2D (VIB-Ghent University, Ghent, Belgium) under the regulation of the CaMV35S promoter. The resultant vector (pB7WG2D-OsBBX14) was introduced into *Agrobacterium tumefaciens* GV3101 using the freeze-thaw method. *Arabidopsis* Col-0 wild-type plants were transformed with this construct using the floral dipping method. Transformants were grown in soil under a 16-h light/8-h dark regimen at 22 °C. Transgenic *Arabidopsis* plants were selected by spraying with 0.3% BASTA. Homozygous T_3_ lines were selected for further analysis.

### 4.6. Measurement of Hypocotyl Length and Total Anthocyanin and Chlorophyll Contents

The seeds of wild-type and transgenic *Arabidopsis* plants were sown on Murashige and Skoog (MS) medium, subjected to cold treatment at 4 °C for 3 days, then grown for 7 days under LD conditions at 22 °C. The hypocotyl lengths of the *Arabidopsis* seedlings were measured using the free software ImageJ (http://imagej.nih.gov/ij).

The seedling anthocyanin content was determined following the method described by Lim et al. [46]. Briefly, 50 *Arabidopsis* seedlings were ground and incubated in 600 μL extraction buffer (methanol containing 1% HCl) for 6 h at 4 °C with moderate shaking. Next, 200 μL water and 200 μL chloroform were added and the samples were centrifuged at 14,000 rpm for 5 min at 4 °C to sediment the plant material. After centrifugation, the absorbance of the supernatant was measured at 530 nm (A_530_) and 657 nm (A_657_) using a microplate reader. The total anthocyanin content was quantified using the following equation: A_530_ − 0.25A_657_. All samples were measured in triplicate, and three independent biological replicates were performed.

The seedling chlorophyll content was determined using the method described by Inskeep and Bloom [47]. Briefly, 100 µg of *Arabidopsis* seedlings were ground in liquid nitrogen and added to 1 mL of 80% acetone to extract their chlorophyll. After centrifugation at 12,000 rpm for 5 min at 4 °C, the absorbance of the supernatant was recorded at 664 nm (A_664_) and 647 nm (A_647_) using a microplate reader. The chlorophyll content was determined using the following equations: chlorophyll A (µg/mL) = 12.70A_664_ − 2.79A_647_; chlorophyll B (µg/mL) = 20.70A_647_ − 4.62A_664_. The total amount of chlorophyll was calculated by adding the contents of chlorophyll A and chlorophyll B.

### 4.7. Transient Expression Assay Using a DEX-Inducible Activation System in Rice Protoplasts

To investigate the targets of OsBBX14, the full-length cDNA of *OsBBX14* was fused into the pTr-GR vector, which contained both the CaMV35S promoter and the *GR* coding sequence. To construct the pTr-OsBBX14-GR vector, the *OsBBX14* sequence except its stop codon was amplified using the primer set pTr-OsBBX14-F/R and ligated into the *Xba*I-digested pTr-GR vector using an In-Fusion HD Cloning Kit (Takara).

The *OsBBX14-GR* construct was introduced into rice leaf protoplasts, as previously described [45]. For rice protoplast preparation, surface-sterilized rice seeds were grown on half-strength MS (supplemented with 1% sucrose, 0.4% phytagel and adjusted to pH 5.8) under dark conditions for 10 days and transferred to continuous light conditions for 3 days. A bundle of rice seedlings were chopped and dipped into enzyme solution (1.5% cellulose R-10, 0.75% macerozyme R-10, 0.6 M mannitol, 10 mM MES at pH 5.7, 0.1% BSA, 3.4 mM CaCl_2_, 5 mM β-mercaptoethanol, and 50 μL·mL^−1^ ampicillin) and incubated for 3–4 h in the dark with gentle shaking (50 rpm). The enzyme solution containing protoplasts was diluted with three volumes of W5 solution (0.1% glucose, 0.9% NaCl, 2 mM MES, 0.08% KCl, and 125 mM CaCl_2_ at pH 5.65) and filtrated through 145-μm mesh into 50-mL conical tubes for removing undigested stem tissues. After centrifugation at 100× *g* for 10 min at 28 °C, the collected protoplasts were re-suspended in 4 mL W5 solution, and then resuspended protoplasts were floated on 5 mL 22% sucrose to separate burst protoplasts. After centrifugation, intact protoplasts were collected and washed with W5 solution, the protoplasts were resuspended in MMg solution (600 mM mannitol, 15 mM MgCl_2_, and 5 mM MES at pH 5.65). For transfections, 300 μL protoplasts (2 × 10^6^ protoplasts/mL) were mixed with plasmid constructs and 330 μL PEG solution (400 mM mannitol, 100 mM Ca(NO_3_)_2_, and 40% PEG-6000). The mixture was incubated for 30 min at 28 °C. After incubation, W5 solution was added stepwise for dilution the PEG solution. Protoplasts were collected by centrifugation at 100× *g* for 10 min at 28 °C. After centrifugation, the protoplast pellet was resuspended in W5 solution for the further treatment.

To activate *OsBBX14* expression, the protoplasts were treated with 10 μM DEX for 4 h. The control protoplasts were mock-treated with the same concentration (0.01%) of ethanol used to dissolve DEX. To inhibit the synthesis of new proteins, 2 μM of the protein synthesis inhibitor CHX was added 30 min before the addition of DEX [27]. After the treatments, the protoplasts were harvested for qPCR analysis. The expression levels of each gene in the control protoplasts without DEX treatment was set to 1, and three biological replications were used. The gene-specific primers used for qPCR analysis are listed in Table S1.

### 4.8. Transactivation and Y2H Assays

To generate the *OsBBX14* BD constructs, complete and partial regions of the *OsBBX14* coding sequence were individually amplified using specific primer sets (Table S1). The amplified fragments were cloned into pGBKT7 vectors harboring the GAL4 DNA-binding domain (Takara) using an In-Fusion HD Cloning Kit (Takara). The individual BD constructs were transformed into the yeast strain AH109, following the manufacturer’s instructions (Takara). The transformed yeast cells were grown on SD media lacking Trp and were replicated on SD media lacking Trp, His, and Ade containing X-α-gal for color development. After two days in darkness at 30 °C, the plates were photographed.

To examine the interaction between OsBBX14 and OsHY5, three BD constructs (OsBBX14N1, OsBBX14N2, and OsBBX14N3) were selected, which were found to have no autoactivation activity in yeast. To generate the OsHY5 activation-domain (AD) constructs, complete and partial regions of the *OsHY5* coding sequence were individually amplified using specific primer sets (Table S1) and cloned into pGADT7 harboring the GAL4 AD. The AD and BD constructs were cotransformed into the yeast strain MaV203, following the manufacturer’s instructions (Takara). Yeast strains were selected on SD medium lacking Trp and Leu and were replicated on SD medium lacking Trp, Leu, and His supplemented with 10 mM 3-AT, a competitive inhibitor of the *HIS3* gene product. After two days in the dark at 30 °C, the plates were photographed.

## Supplementary Materials

Supplementary materials can be found at http://www.mdpi.com/1422-0067/19/8/2190/s1.
