# Supplementary material for: A Rice B-Box Protein, OsBBX14, Finely Regulates Anthocyanin Biosynthesis in Rice
Source: Int J Mol Sci. 2018 Jul 27;19(8):2190. doi: 10.3390/ijms19082190 (PMC6121638; doi:10.3390/ijms19082190)
Supplement: Supplementary file 1 [file ijms-19-02190-s001.pdf]

Table S1. Gene specific primers used in this study.

Usage	Primer Name	Primer Sequence
Gene expression during rice seed development stages	OsBBX14-F1	GCCTCCTCCTCCACCATATT
	OsBBX14-R1	AAGAACCCGAGCTCCTCCT
	OsHY5-F1	GCGTACATGAGTGAGCTGGA
	OsHY5-R1	CGTTTGCAGGGTAGAGAGC
	OsC1-F	CTGGAACTTGTGTGCGTGAC
	OsC1-R	CCCGCAACTGCACTTAAAAT
	OsB2-F	CCGAGAGAAGCTCAACGAGA
	OsB2-R	TGCAAGTATGGATGCCTTGT
	OsTTG1-F	CAGCTTACCCGGAGTGGATA
	OsTTG1-R	CCAAGGCTTCAAGTCGAAC
	OsUBI-F	GAAGTAAGGAAGGAGGAGGA
	OsUBI-R	AAGGTGTTCAGTTCCAAGG
Subcellular localization	P326_OsBBX14-F	CACGGGGGACTCTAGAATGTCGCCTCCTCCTCCA
	P326_OsBBX14-R	CCATGGATCCTCTAGATTGCCTCCGGCGTTTGGA
Transactivation assay	pGBKT7_OsBBX14-L-F	CATGGAGGCCGAATTCATGTCGCCTCCTCCTCCA
	pGBKT7_OsBBX14-L-R	GGATCCCCGGGAATTCTTGCCTCCGGCGTTTGGA
	pGBKT7_OsBBX14-C1-F	CATGGAGGCCGAATTCATGTCGCCTCCTCCTCCA
	pGBKT7_OsBBX14-C1-R	GGATCCCCGGGAATTCGGAGGTGGTGGCCGGGAA
	pGBKT7_OsBBX14-C2-F	CATGGAGGCCGAATTCACCGGCGTGCAGGTCGGG
	pGBKT7_OsBBX14-C2-R	GGATCCCCGGGAATTCTTGCCTCCGGCGTTTGGA
	pGBKT7_OsBBX14-C3-F	CATGGAGGCCGAATTCGCCGACTCGCCGGCCGCG
	pGBKT7_OsBBX14-C3-R	GGATCCCCGGGAATTCTTGCCTCCGGCGTTTGGA
Expression analysis in trnasgenic Arabidopsis plants	AtHY5-F	GAGAACCAGATGCTTAGACA
	AtHY5-R	AATCCTAACCTTCTCCTTATTCAC
	AtTTG1-F	TTGTTCTGGTGGTGATGATAC
	AtTTG1-R	CAATCAGGCTGCGAAGAA
	AtTT8-F	TCTAATGGAGGAAGGTGGAA
	AtTT8-R	AACGATGATTGGATGTAAGAAGA
	AtEGL3-F	GATACAGAGACACGGATAACG

	AtEGL3-R	CTCCTACATTCACATCACTCC
	AtMYB12-F	TCTCAACTACGACCACCAA
	AtMYB12-R	ATGCCAAAGATTATTATCACTACCT
	AtPAP1-F	GAAGCGACGACAACAGAA
	AtPAP1-R	GAAGCGACGACAACAGAA
	AtMYB113-F	ATCTTGTTCTTCGCCTTCATAA
	AtMYB113-R	CATCGTTCATCGTGCTTCT
	AtMYB114-F	TCTTCTTCTTCGTCTTCATAAGC
	AtMYB114-R	GGGTGTTCCAGTAGTTCTTG
	AtEFlα-F	GCCACACCTCTCACATTG
	AtEF1α-R	TACCAGCGTCACCATTCT
qPCR analysis in rice protoplast	OsC1-F	CTGGAACTTGTGTGCGTGAC
	OsC1-R	CCCGCAACTGCACTTAAAAT
	OsB2-F	CCGAGAGAAGCTCAACGAGA
	OsB2-R	TGCAAGTATGGATGCCTTGT
	OsTTG1-F	CAGCTTACCCGGAGTGGATA
	OsTTG1-R	CCAAGGCTTCAAGTCGAAC
	OsHY5-F2	AGCTGCTCCCTTTTGATTGA
	OsHY5-R2	GGTGCATAGCCAAGAATGGT
	OsBBX14-F2	AATAGCTCGAGACGCTGACG
	OsBBX14-R2	ACAACCAACCAACCAACCAC
	OsUBI-F	GAAGTAAGGAAGGAGGAGGA
	OsUBI-R	AAGGTGTTCAGTTCCAAGG
Yeast two hybrid	pGBKT7_OsBBX14-L-F	CATGGAGGCCGAATTCATGTCGCCTCCTCCTCCA
	pGBKT7_OsBBX14-L-R	GGATCCCCGGGAATTCTTGCCTCCGGCGTTTGGA
	pGBKT7_OsBBX14-C1-F	CATGGAGGCCGAATTCATGTCGCCTCCTCCTCCA
	pGBKT7_OsBBX14-C1-R	GGATCCCCGGGAATTCGGAGGTGGTGGCCGGGAA
	pGBKT7_OsBBX14-C2-F	CATGGAGGCCGAATTCACCGGCGTGCAGGTCGGG
	pGBKT7_OsBBX14-C2-R	GGATCCCCGGGAATTCTTGCCTCCGGCGTTTGGA
	pGBKT7_OsBBX14-C3-F	CATGGAGGCCGAATTCGCCGACTCGCCGGCCGCG
	pGBKT7_OsBBX14-C3-R	GGATCCCCGGGAATTCTTGCCTCCGGCGTTTGGA
	pGADT7_OsHY5-L-F	GGAGGCCAGTGAATTCATGCAACGAGATCACCGG
	pGADT7_OsHY5-L-R	CACCCGGGTGGAATTCCTAGCTGTCTCCGCCGGC

pGADT7_OsHY5-N-F	GGAGGCCAGTGAATTCATGCAACGAGATCACCGG
pGADT7_OsHY5-N-R	CACCCGGGTGGAATTCCAGCAATCTTTTGAGGC
pGADT7_OsHY5-C-F	GGAGGCCAGTGAATTCAGGAACCGGGTGTCAGCG
pGADT7_OsHY5-C-R	CACCCGGGTGGAATTCCTAGCTGTCTCCGCCGGC

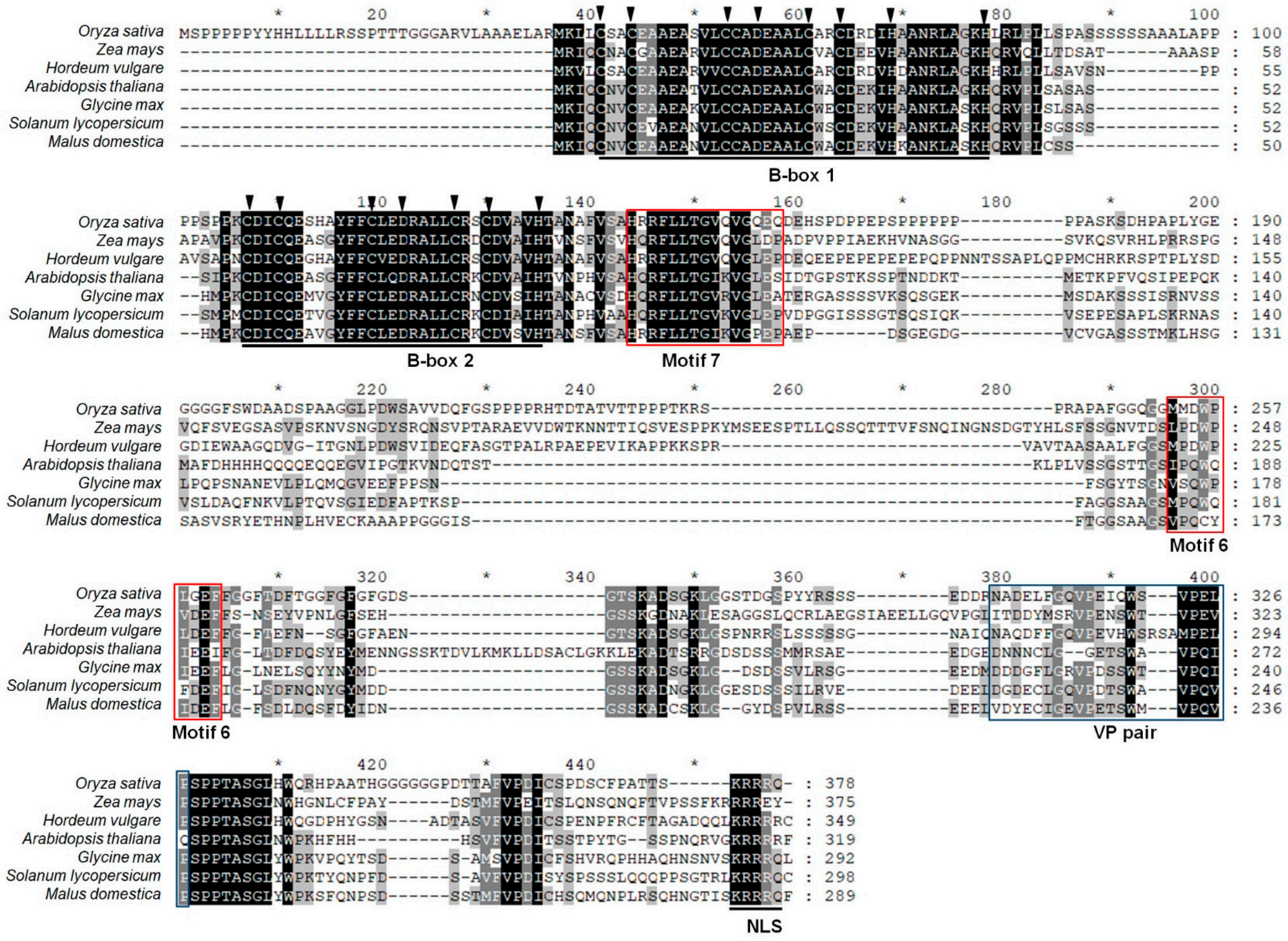

Figure S1. Comparison of deduced amino acid sequences of subfamily IV of the BBX proteins from various plants species. The solid lines indicate highly conserved zinc finger domains, B-box domain 1 and 2. The red boxes indicate conserved motif (motif 6 and 7) among subfamily IV BBX proteins, and the VP pair and the nuclear localization signals (NLS) are shown in the blue box and on the short solid line, respectively. .
